# Predictors of five-year relapse rates of youths with substance abuse who underwent a family-oriented therapy program

**DOI:** 10.1186/s12991-020-00269-4

**Published:** 2020-03-10

**Authors:** Yu-Han You, Shing-Fang Lu, Chih-Pu Tsai, Mei-Yen Chen, Chin-Yin Lin, Mian-Yoon Chong, Wen-Jiun Chou, Yi-Syuan Chen, Liang-Jen Wang

**Affiliations:** 1 Department of Child and Adolescent Psychiatry, Kaohsiung Chang Gung Memorial Hospital and Chang Gung University College of Medicine, 123, Ta-Pei Road, Niao-Sung District, Kaohsiung City, 83301 Taiwan; 2 Taiwan Kaohsiung Juvenile and Family Court, Kaohsiung, Taiwan; 3 Department of Psychiatry, Kaohsiung Chang Gung Memorial Hospital and Chang Gung University College of Medicine, Kaohsiung, Taiwan

**Keywords:** Substance abuse, Psychotherapy, Family, Juvenile delinquency, Relapse, Predictor

## Abstract

**Background:**

Substance abuse among young people has become a serious public health problem for years. The risk of relapse among illicit drug use is essential for developing adequate substance reuse prevention policies. The purpose of the current study is to investigate the potential predictor in long-term relapse rates among young patients that underwent a family-based treatment program.

**Methods:**

To perform this study, 103 young patients with substance use (mean age: 16.2 years, 78.6% male) were referred to participate in a 10-week family-based treatment program. At the beginning and at the end of the treatment, the patients were required to fill out the Chinese Craving Beliefs Questionnaire (CCBQ), the Adolescents’ Behavior-problem Scale (ABS), and the Family APGAR. Furthermore, the patients’ caregivers had to fill out the Family APGAR, the 12-item version of the Chinese Health Questionnaire (CHQ), and the Parenting Stress Index (PSI). All patients were followed up for 5 years in order to observe their long-term outcomes regarding substance use relapse.

**Results:**

During the 10-week family-oriented programs, the CCBQ scores, the CHQ scores and the Child-domain of PSI significantly decreased. Better changes in patients’ behavioral problems during the treatment program predicted a lesser likelihood of substance use relapse in the subsequent 5 years. Furthermore, methamphetamine or 3,4-methylenedioxy-methamphetamine use and living in single-parent families were two factors associated with higher relapse rates.

**Conclusions:**

The changes in patients’ behavioral problems during the treatment program may serve as a predictor of substance use relapse over the subsequent 5 years. This study’s findings provide insight about substance use prevention and serve as a reference for policy-making.

## Background

Substance abuse among young people has become a serious public health problem for years [1]. In the United States alone, 75.6% of young people under the age of 18 have admitted to use addictive substances (such as cigarettes, alcohol, marijuana and/or cocaine) at least once [2, 3]. Recently, drug abuse among Taiwanese adolescents has also been on the rise [4, 5], with ketamine, ecstasy and methamphetamine being the most popular illicit drugs [6–8]. Substance abuse can lead to serious and harmful complications in young people's lives, including physical illnesses, cognitive impairments, and issues with academic and occupational function, which can cause social burdens and even death in some cases [9, 10]. Therefore, understanding the risk of relapse of illicit drugs is critical to developing an appropriate policy for the prevention of substance reuse.

Young people with drug abuse problems are often considered juvenile delinquents and may be sentenced to probation, rehabilitation or detoxification [11]. To solve this problem, the Kaohsiung Junior Family Court in Taiwan and the Kaohsiung Chang Gung Memorial Hospital have been cooperating since 2010 on a specific treatment plan for drug addicts and their caregivers. Court orders stipulate that minors arrested for drug abuse must undergo treatment procedures at the hospital. In this research, the corresponding programs included 10 sessions of weekly psychotherapy courses based on the motivational enhancement principle [12] and 10 sessions of weekly parenting-skill training courses for caregivers [13–15]. Detailed information on this family-oriented treatment plan and its effectiveness for young drug addicts and their caregivers has been provided in our previous studies [16, 17].

Our previous research results showed that after treatment, the frequency of substance cravings by the young participants decreased, while the family function, as perceived by the participating caregivers, increased significantly. The improvement in caregiver's perception of family function is positively correlated with the improvement of the caregiver's health status [16]. Moreover, it was also found that family-oriented treatments might be a more effective option to prevent adolescents from relapsing into substance abuse. Compared with individuals who were subject to the standard supervision by the court, those receiving MEP showed higher school attendance rates or better social outcomes in the follow-up period [17]. Despite this, it is still unclear whether the changes in those treated young people can be used to predict their subsequent substance-use relapse.

The young patients who completed the family-oriented treatment program also received long-term follow-up in the justice system, which enables us to determine whether the changes during the treatment program are associated with subsequent substance-use relapse. Therefore, the purpose of the current study is to investigate potential predictors of long-term relapse rates among young patients that have participated in the family-based treatment program.

## Methods

### Research participants

This research was approved by the Institutional Review Board of Chang Gung Memorial Hospital (IRB102-0771A3). The judges of Taiwan’s Kaohsiung Juvenile and Family Court required underage individuals that have been arrested for substance use to participate in the treatment program in Kaohsiung Chang Gung Memorial Hospital. In total, 103 young participants who attended treatment at the hospital and their primary caregivers were recruited consecutively between July 2011 and December 2013. The inclusion criteria were as follows: (1) aged between 12 and 18 years old; (2) having illicit drug use; and (3) at least one of their caregivers could attend the treatment program. The exclusion criteria included (1) intellectual disability, (2) having apparent psychotic symptoms, and (3) having first-class illicit drug use (i.e., heroin, morphine, or cocaine).

During this research, the 103 patients were ordered to attend 10 sessions of weekly out-patient drug abuse treatment program while their caregivers were required to participate in 10 sessions of weekly parenting-skill training program. Both programs were held at the Kaohsiung Chang Gung Memorial Hospital. Before taking part in this research, each participant submitted a signed written informed consent, while the researchers emphasized whether to take part in this research or not would never affect his or her legal status and that all personal information provided would be kept strictly confidential. Details of treatment protocols and research procedures have been demonstrated elsewhere [16, 17].

### Motivational enhancement psychotherapy program for young people

Young participants received 10 treatment sessions on a weekly basis. Led by two experienced psychologists from the hospital, the group relapse prevention program, which was basically constituted of motivational enhancement ideas, accommodated about eight participants in each session lasting 120 min with a 10-min break. The aim of such a group counseling meeting was to evoke the young participants’ motivations for a change by identifying the causes for their drug addiction, the reasons for their abstinence from substance abuse, their concerns about substance use and their perception of illicit drugs in their current and long-term lives. Having developed a rapport with the participants, the psychologists were able to learn how illicit drugs fitted into their lives. Feedback was the main counseling technique used during the meeting, in which the psychologists asked questions and the participants reflected their past in the hope to prompt the latter’s self-motivational statements [18]. The focus of the treatment program was to identify the situations which had put the young participants at high risk for drug abuse, to enhance their motivation to abstain from substance abuse and to develop coping strategies to prevent relapse.

### Parenting skills training program for caregivers

The parenting skills training program also included 10 weekly 120-min sessions, conducted by two senior counseling psychologists or social workers appointed by the court. The therapists first shared common legal knowledge to alleviate the concerns and helplessness of the caregivers and then handled their emotional reactions to their teens, such as shock, anger and frustration. Helping the caregivers check out their current relationships and their communication skills with their teenagers, the therapists were able to guide the caregivers to judge how negative their family relationships were and how they could change their own behaviors and attitudes so as to improve their daily home environment and atmosphere. Not only did the therapists provide the caregivers with new ways to engage with their adolescents, but they also taught the caregivers how to solve such issues as their adolescents separating from them in a non-normative manner [19]. The purpose of this training program was to teach those caregivers more effective skills to deal with the drug abuse problems caused by their youths.

### Research procedures and measures

**Fig. 1:**
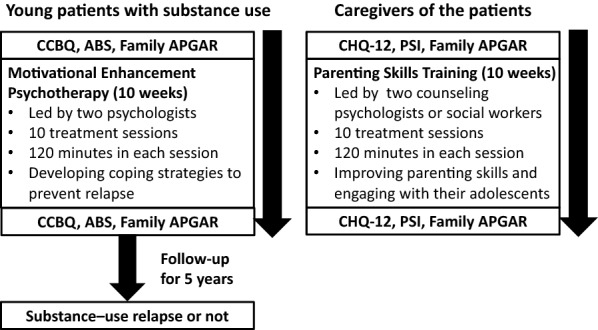
The main points of therapy and flowchart of research procedures. *CCBQ* the Chinese Craving Beliefs Questionnaire, *ABS* The Adolescents’ Behavior-problem Scale, *CHQ-12* The 12-item version of the Chinese health questionnaire, *PSI* The Chinese version of the Parenting Stress Index

Figure 1 illustrates the main points of therapy and flowchart of research procedures. At the beginning of the treatment courses, the information on sociodemographic characteristics of participating patients (e.g., categories of substances being used, history of previous convictions, family status, and academic or social status) was provided by Taiwan’s Kaohsiung Juvenile and Family Court. The young patients were asked to fill out the Chinese Craving Beliefs Questionnaire (CCBQ), the Adolescents’ Behavior-problem Scale (ABS) and the Family APGAR at both the first and last treatment sessions (10 weeks later). The patients’ caregivers were required to complete the Family APGAR, the 12-item version of the Chinese health questionnaire (CHQ-12) and the Chinese version of the Parenting Stress Index (PSI) at the first session and the last session of treatment as well.

The CCBQ was adapted from a craving beliefs questionnaire (CBQ) developed by Wright [20]. This questionnaire, consisting of 10 items, measures beliefs and understandings of substance cravings and is answered with a 4-point Likert scale (from total disagreement (1) to total agreement (4)). The higher the total score, the higher the substance craving. With acceptable reliability and effectiveness, the CCBQ is suitable as a research tool for assessing the substance craving beliefs [21].

The ABS is a self-administered questionnaire used to measure the emotional disturbance of the young patients. The ABS consists of 50 items, all of which are answered with a 6-point Likert scale. Divided into five categories, namely self-awareness, physical and mental development, school life, interpersonal relationships and family life, the ABS can be used to give a composite score and has good reliability and validity as a questionnaire [22].

Family APGAR has been widely used to measure family’s well-being [23]. It involves the following five items: degree of adaptation, partnership, growth, affection and resolution in the family. Each item is answered using a 3-point Likert scale ranging from 0 (low satisfaction) to 2 (high satisfaction). The total score ranges from 0 to 10. The higher the score, the better the family function. The Chinese version of the questionnaire has sufficient internal reliability and validity [24].

The CHQ-12, a 12-item self-reporting questionnaire, is a modified version of the General Health Questionnaire [25]. This tool has been widely used in research to identify subjects in primary care and those in a community setting who have minor psychiatric disorders. A 4-point Likert scale (0 = not at all, to 3 = more than usual) is provided as a response format to analyze the conditions during the preceding 2 weeks (score range = 12–36). The higher the score, the worse the health condition. The questionnaire has good reliability and validity [26].

The PSI is a standardized Chinese version of the original 120-item questionnaire used to measure the elements of parental function [27]. The PSI Parent Domain Scale contains 54 items and consists of seven subscales. The PSI Child Domain Scale contains 47 items and has six subscales. The parent and child domains are added together to get a total score and a derived raw-to-percentile score. The patients’ caregivers rated their level of agreement with these items on a 5-point Likert scale. The higher the score, the greater the level of parenting stress [28].

### Follow-up for substance-use relapse

Upon completing the treatment courses, all the participants were reverted to court supervision and probation again, including notifying the protection officers of their academic, social, and living status about once every month. During the follow-up period, the patients were required to provide urine samples, which the judges or protection officers would test to decide at their own discretion whether or not drug residues were present. If a test result was positive, the court would give the youth in question a hearing and then sentence him or her to take reformatory education or to be confined in the detoxification unit of the detention center. The outcome of this research was concerned with substance-use relapse during a 5-year follow-up period.

### Statistical analysis

We analyzed the data in this study using the statistical software package SPSS, version 21.0 (SPSS Inc., Chicago, IL, USA). Variables are presented as either mean ± standard deviation (SD) or frequency. Paired-t test was adopted to examine any changes in the measures for either the patients or their caregivers throughout the 10-week treatment program. Receiver operating characteristic (ROC) curves and the area under the curve (AUC) were used to evaluate whether the treatment effectiveness served as predictors of patients’ relapse during the 5-year follow-up period.

The completion date of the family-oriented program was set as the index date and was used to calculate risk over time. As for survival analysis, the time function was defined as the number of days from the index date to the end of the period for those patients who had no other instance of substance use to that point or to the date of relapse if such relapse occurred before the end of the follow-up period. We controlled for socio-demographic variables to develop a Cox regression model to estimate the treatment effects on relapse. Adjusted hazard ratios (aHR) were calculated with 95% confidence intervals (CI). We considered a two-tailed *p*-value less than 0.05 statistically significant.

## Results

The mean age of the 103 young patients with substance use was 16.2 ± 1.0 years, with 81 (78.6%) of them being males (Table 1). Regarding the school or social status of these patients, 35 (34.0%) were still attending school; 34 (33.0%) had been suspended or dropped out from school; and 34 (33.0%) were employed. Regarding family status, 47 (45.6%) lived in double-parent families; 42 (40.8%) lived in single-parent families; and 14 (13.6%) had another situation (raised by grandparent(s)). Of the 103 substance-use patients, 74 (71.8%) of them used ketamine and 29 (28.2%) of them used 3,4-methylenedioxy-methamphetamine (MDMA) or methamphetamine. Twenty-seven patients had previous conviction records related to substance use.

**Table 1:** Characteristics of young patients with substance abuse (*N* = 103) participating in a weekly 10-week out-patient treatment program

Variables	Mean or *N*	SD or %
Age (years)	16.2	1.0
Sex		
Female	22	21.4
Male	81	78.6
School status or social status		
Residence	35	34.0
Suspension or dropout	34	33.0
Employed	34	33.0
Family status		
Double-parent families	47	45.6
Single-parent families	42	40.8
Grandparent(s)	14	13.6
Substance in use		
Ketamine	74	71.8
MDMA or methamphetamine	29	28.2
Previous conviction record at baseline		
Without	76	73.8
With	27	26.2
Relapse		
Yes	39	37.9
No	64	62.1

Of the 103 patients that participated in this study, 97 (94.2%) completed the 10-week treatment program and assessment. Table 2 shows the changes in the patients’ measures and those of their caregivers from the baseline to the endpoint of the follow-up period. During the 10-week family-oriented programs, the CCBQ scores of the patients decreased significantly (*t* = 4.510, *p* < 0.001), while the CHQ scores (*t* = 3.463, *p* = 0.001) and the Child-domain of PSI (*t* = 2.276, *p* = 0.025) filled in by their caregivers also significantly decreased. Nevertheless, the ABS scores, Family APGAR scores, and Parent-domain PSI scores revealed no significant changes over the 10-week period (*p* > 0.05).

**Table 2:** Measures of the patients and their caregivers at the baseline and 10 weeks later through a family-oriented out-patient treatment program for youths with substance use disorder

	Baseline	10 weeks later	Statistic value (*t*)	*P*-value
Measures of youths				
Chinese Craving Beliefs Questionnaire	13.6 ± 4.5	11.5 ± 2.7	4.510	< 0.001*
Adolescents’ Behavior-Problem Scale	92.4 ± 23.3	94.1 ± 28.1	-0.706	0.482
Family APGAR-Adolescents	6.5 ± 2.7	6.5 ± 3.0	-0.077	0.939
Measures of youths’ caregivers				
Family APGAR-Caregivers	5.7 ± 2.9	5.6 ± 2.8	0.342	0.733
Chinese health questionnaire	2.6 ± 2.5	1.7 ± 2.3	3.463	0.001*
Parenting Stress Index-Parent domain	140.0 ± 22.6	136.1 ± 24.2	1.751	0.083
Parenting Stress Index-Child domain	124.0 ± 22.4	119.1 ± 25.8	2.276	0.025*

*p < 0.05; ***p < 0.001

**Fig. 2:**
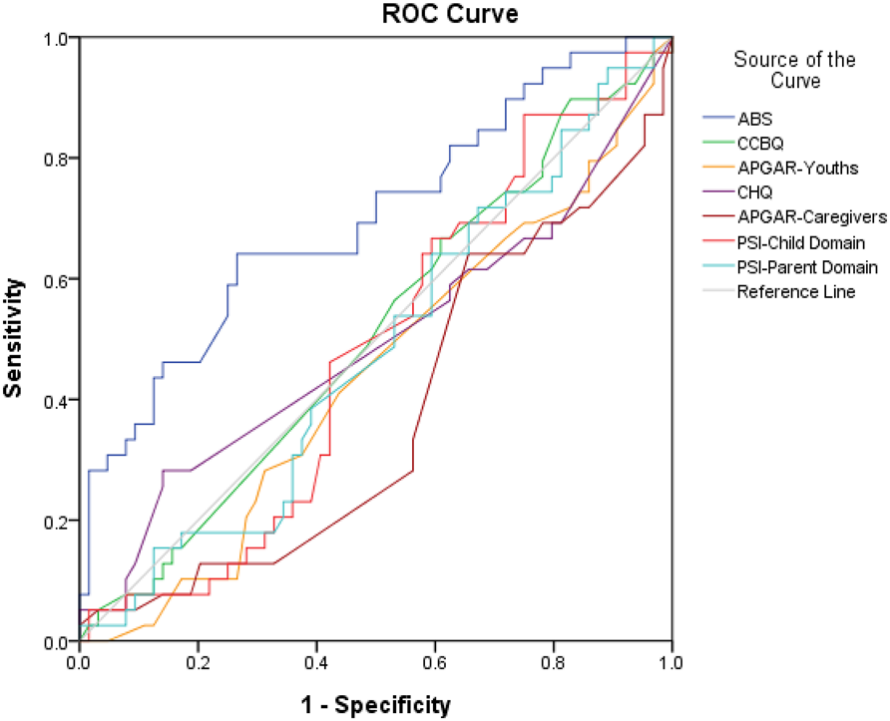
The ROC curve of using the proportion of changes in the patients’ measures and those of their caregivers to predict patients’ relapse of substance use. *CCBQ* The Chinese Craving Beliefs Questionnaire, *ABS* The Adolescents’ Behavior-problem Scale, *CHQ-12* The 12-item version of the Chinese health questionnaire, *PSI* The Chinese version of the Parenting Stress Index. The proportion of changes in the youths’ ABS scores (AUC = 0.70, *p* = 0.001) and Family APGAR scores rated by caregivers (AUC = 0.37, *p* = 0.031) significantly differentiated youths with relapse and without relapse

Of the 103 substance-using patients, 39 (37.9%) relapsed within a follow-up of 5 years. We used ROC curves to investigate whether the measures during the 10-week treatment program were able to significantly predict their subsequent relapse. Figure 2 shows the ROC curve of using the proportion of changes in the patients’ measures and those of their caregivers to predict patients’ relapse of substance use. We found that the proportion of changes in the youths’ ABS scores (AUC = 0.70, *p* = 0.001) and Family APGAR scores rated by caregivers (AUC = 0.37, *p* = 0.031) significantly differentiated patients with relapse and without relapse. However, the other measures did not significantly predict patients’ relapse of substance use.

The Cox proportional hazard models of the risk of relapse in the follow-up period are shown in Table 3. After controlling for socio-demographic variables, the changes in ABS scores were still able to significantly predict substance use relapse (aHR = 10.51, 95%CI 1.70–65.01, *p* = 0.011). Compared to the patients that used ketamine, the patients who used 3,4-methylenedioxy-methamphetamine (MDMA) or methamphetamine had a significantly higher risk of relapse (aHR = 2.27, 95%CI 1.02–5.06, *p* = 0.046). Furthermore, the patients’ family status also significantly predicted the relapse rates. Compared to subjects living in double-parent families, youths who lived in single-parent families were more likely to relapse in substance abuse during the study period (aHR = 2.77, 95%CI 1.24–6.21, *p* = 0.013).

**Table 3:** Risk of relapse after the index substance use for related variables estimated by Cox proportional hazards model

Variables	Cox regression model	
	aHR (95% CI)	*P*-value
Age (years)	0.86 (0.60–1.21)	0.383
Sex		
Male	1	
Female	0.43 (0.14–1.34)	0.145
Substance use		
Ketamine	1	
MDMA or methamphetamine	2.27 (1.02–5.06)	0.046*
Previous conviction record		
Without	1	
With	1.37 (0.55–3.42)	0.501
Academic or social status		
Attending school	1	
Employed	0.48 (0.18–1.27)	0.138
Dropout and unemployed	0.96 (0.39–2.37)	0.925
Family status		
Double-parent families	1	
Single-parent families	2.77 (1.24–6.21)	0.013*
Grandparent(s)	0.58 (0.12–2.74)	0.489
Change of behavior-problem	10.51 (1.70–65.01)	0.011*
Change of family APGAR-caregivers	0.75 (0.38–1.49)	0.414

*aHR* adjusted hazard ratio, *95% CI* 95% confidence interval

*p < 0.05

## Discussion

This study provides the results of a family-oriented group treatment program that addressed both substance-using young patients and their caregivers. We found that changes in patients’ behavioral problems during the treatment program may serve as a predictor of substance use relapse during the subsequent 5 years. Furthermore, the categories of substance use and family status of the patients were also associated with their relapse.

In the 10-week group motivation-enhanced psychotherapy, whose main goal was to encourage patients to stop using drugs, the young participants' substance craving beliefs (the main result of this research) decreased significantly, suggesting that the treatment plan had produced positive effects. In addition, the CHQ scores and the Child-domain of PSI filled in by caregivers were significantly reduced as well, indicating that the caregivers who had felt helpless and frustrated at the beginning might have significantly improved along with the treatment program to have developed a better mental health and ability to face and interact with their children. The Child Domain here is defined as those qualities demonstrated by children which make it difficult for their parents to fulfill their parenting roles [29], and the training program was meant to help the caregivers reform their relationships with their teenagers and explore better strategies to get along with their children. To this end, the therapists worked together with the caregivers to enhance their parenting, communication, and problem-solving skills so as to make them more capable of fulfilling their parenting roles [30, 31].

The 5-year relapse rate among the young patients with substance use was 38%. A study of adolescent crack users revealed high rates of relapse of 65.9 and 86.4% in the first and third months, respectively [32]. Our previous cohort revealed that the 2-year relapse rate reached 33.1% among substance-use adolescents [17]. This finding suggests that breaking the drug addiction cycle for substance-using adolescents remains a challenge [33]. Notably, after the 10-week program for parenting skills training, although the ABS scores did not change significantly, the changes of ABS were the most significant index for predicting the 5-year relapse of substance use, and an increased trend of ABS was linked to a higher risk of relapse. The ABS is a self-administered questionnaire that measures patients’ emotional disturbance; if patients have a greater improvement in emotional disturbance, they are less likely to reuse a substance. Said finding supports that adolescents with substance use disorder may have high rates of depression or anxiety, and these unreleased emotional disturbances may be subsequently related to substance use [34]. Also other psychiatric disorders, such as post-traumatic stress disorder (PTSD), have been reported to be related to emotional disturbance. PTSD may be characterized by the presence of maladaptive behaviors as substance abuse, particularly in male young patients [35]. The aforementioned findings give us insight about the predictor in this treatment program and remind clinicians that the emotional status of youths warrants special attention in order to prevent patients from relapsing with substance use [32].

It was found that compared to patients using ketamine, those using MDMA or methamphetamine had 2.65 times higher relapse rates. This finding was consistent with our previous long-term follow-up study [36]. The difference in relapse risk was associated with the pharmacological properties of ketamine and stimulants. For example, the users of methamphetamine or MDMA might have had greater drug craving behaviors than ketamine users, thus leading to a higher relapse rate. Relative to the users of stimulant drugs, the ketamine users might have been in the earlier stage of substance use and might have had a greater potential to achieve abstinence from drug abuse. In addition, young addicts in single-parent families were more prone to drug relapse during the study period than those living in double-parent families. Early onset drug users might have been associated with a variety of psychosocial problems, such as behavioral patterns, family systems, peer relationships, and job adjustments [37]. Likewise, parent–child attachment, monitoring and parenting methods were identified as important factors in preventing adolescents from drug abuse [38]. Our research also revealed that living in a double-parent family might have protected young people from further drug use; the reason might be related to greater parent–child attachment and stricter discipline in a double-parent family.

This research has several limitations. First, the study was conducted in a non-comparative, non-randomized and open-label manner. All the measures in this research were self-administered; as a result, they might have been affected by socially desirable responses and motivation bias. Second, this study lacks a control group for confirming the effect of the intervention program. Third, some critical variables potentially related to substance use relapse were not recorded in this study, such as alcohol consumption, age of first onset or psychiatric comorbidities (anxiety disorders, depressive disorder or personality disorders) [39]. In addition, we did not have detailed information about the subjects who refused to participate. Fourth, ABS scores did not change significantly during the 10-week treatment. Nevertheless, ABS changes were the most significant index for predicting a 5-year relapse of substance use, which may represent that youths with substance use had a great variety of behavioral problems. Finally, this study’s sample size was small, and a validation group is warranted to verify our findings.

## Conclusion

This study reveals the potential effects of a 10-week treatment program for substance using young patients and their caregivers in Taiwan. We found that changes in patients’ behavioral problems during the treatment program may serve as a predictor of substance use relapse during the subsequent 5 years. This study’s findings provide insight about substance use prevention and serve as a reference for policy-making.

## Data Availability

Data are available from the authors upon reasonable request and with permission of LJW.

## Abbreviations

CCBQ: The Chinese Craving Beliefs Questionnaire
ABS: The Adolescents’ Behavior-problem Scale
CHQ-12: The 12-item version of the Chinese health questionnaire
PSI: The Chinese version of the Parenting Stress Index

## References

[1] Merikangas KR, McClair VL (2012). Epidemiology of substance use disorders. Hum Genet.

[2] Feinstein EC, Richter L, Foster SE (2012). Addressing the critical health problem of adolescent substance use through health care, research, and public policy. J Adolesc Health.

[3] Park MJ, Scott JT, Adams SH, Brindis CD, Irwin CE (2014). Adolescent and young adult health in the United States in the past decade: little improvement and young adults remain worse off than adolescents. J Adolesc Health.

[4] Hsu LY (2014). Ketamine use in Taiwan: Moral panic, civilizing processes, and democratization. Int J Drug Policy.

[5] Chen WJ, Wu SC, Tsay WI, Chen YT, Hsiao PC, Yu YH, Ting TT, Chen CY, Tu YK, Huang JH (2017). Differences in prevalence, socio-behavioral correlates, and psychosocial distress between club drug and hard drug use in Taiwan: results from the 2014 national survey of substance use. Int J Drug Policy.

[6] Chen WJ, Fu TC, Ting TT, Huang WL, Tang GM, Hsiao CK, Chen CY (2009). Use of ecstasy and other psychoactive substances among school-attending adolescents in Taiwan: national surveys 2004–2006. BMC Public Health.

[7] Chen YT, Chen CY, Chen WJ (2011). Comparative epidemiology of betel nut use versus ecstasy use among Taiwanese adolescents: findings from a national survey. Drug Alcohol Depend.

[8] Wang PW, Yen CF (2017). Adolescent substance use behavior and suicidal behavior for boys and girls: a cross-sectional study by latent analysis approach. BMC Psychiatry.

[9] Whiteford HA, Degenhardt L, Rehm J, Baxter AJ, Ferrari AJ, Erskine HE, Charlson FJ, Norman RE, Flaxman AD, Johns N (2013). Global burden of disease attributable to mental and substance use disorders: findings from the global burden of disease study 2010. Lancet.

[10] Ferrari AJ, Norman RE, Freedman G, Baxter AJ, Pirkis JE, Harris MG, Page A, Carnahan E, Degenhardt L, Vos T (2014). The burden attributable to mental and substance use disorders as risk factors for suicide: findings from the global burden of disease study 2010. PLoS ONE.

[11] Das JK, Salam RA, Arshad A, Finkelstein Y, Bhutta ZA (2016). Interventions for adolescent substance abuse: an overview of systematic reviews. J Adolesc Health.

[12] Walker DD, Stephens R, Roffman R, Demarce J, Lozano B, Towe S, Berg B (2011). Randomized controlled trial of motivational enhancement therapy with nontreatment-seeking adolescent cannabis users: a further test of the teen marijuana check-up. Psychol Addict Behav.

[13] McGillicuddy NB, Rychtarik RG, Duquette JA, Morsheimer ET (2001). Development of a skill training program for parents of substance-abusing adolescents. J Subst Abuse Treat.

[14] Liddle HA, Rowe CL, Dakof GA, Ungaro RA, Henderson CE (2004). Early intervention for adolescent substance abuse: pretreatment to posttreatment outcomes of a randomized clinical trial comparing multidimensional family therapy and peer group treatment. J Psychoactive Drugs.

[15] Tanner-Smith EE, Wilson SJ, Lipsey MW (2013). The comparative effectiveness of outpatient treatment for adolescent substance abuse: a meta-analysis. J Subst Abuse Treat.

[16] Wang LJ, Lu SF, Chou WJ, Chong MY, Wang YH, Hsieh YL, Lee YH, Chen C (2015). A family-oriented treatment program for youths with ketamine abuse and their caregivers: a pilot study in Taiwan. Neuropsychiatr Dis Treat.

[17] Wang LJ, Lu SF, Chong MY, Chou WJ, Hsieh YL, Tsai TN, Chen C, Lee YH (2016). A family-oriented therapy program for youths with substance abuse: long-term outcomes related to relapse and academic or social status. Neuropsychiatr Dis Treat.

[18] Cornelius T, Earnshaw VA, Menino D, Bogart LM, Levy S (2017). Treatment motivation among caregivers and adolescents with substance use disorders. J Subst Abuse Treat.

[19] Botzet AM, Dittel C, Birkeland R, Lee S, Grabowski J, Winters KC (2019). Parents as interventionists: addressing adolescent substance use. J Subst Abuse Treat.

[20] Wright FD: Craving beliefs questionnaire. In: Cognitive Therapy of Substance Abuse. Edited by Beck AT, Wright FD, Newman CF, Liese BS. The Guilford Press. NY: 1993: 312.

[21] Chang CW, Huang CW, Wu WH, Wang BE, Liu YL, Shen HC, Lee TS (2011). Psychometric properties of the Chinese craving beliefs questionnaire for heroin abusers in methadone treatment. BMC Psychiatry.

[22] Hagborg WJ (1992). Prevalence and correlates of self-reported depressive mood among seriously emotionally disturbed adolescents. Psychol Rep.

[23] Smilkstein G, Ashworth C, Montano D (1982). Validity and reliability of the family APGAR as a test of family function. J Fam Pract.

[24] Chau TT, Hsiao TM, Huang CT, Liu HW (1991). A preliminary study of family Apgar index in the Chinese. Kaohsiung J Med Sci.

[25] Chong MY, Wilkinson G (1989). Validation of 30- and 12-item versions of the Chinese health questionnaire (CHQ) in patients admitted for general health screening. Psychol Med.

[26] Cheng TA, Williams P (1986). The design and development of a screening questionnaire (CHQ) for use in community studies of mental disorders in Taiwan. Psychol Med.

[27] Weng YS (2003). Parenting stress index.

[28] Yang P, Jong YJ, Hsu HY, Tsai JH (2007). Psychiatric features and parenting stress profiles of subtypes of attention-deficit/hyperactivity disorder: results from a clinically referred Taiwanese sample. J Dev Behav Pediatr.

[29] Loyd BH, Abidin RR (1985). Revision of the parenting stress index. J Pediatr Psychol.

[30] Kamon JL, Stanger C, Budney AJ, Dumenci L (2006). Relations between parent and adolescent problems among adolescents presenting for family-based marijuana abuse treatment. Drug Alcohol Depend.

[31] Hummel A, Shelton KH, Heron J, Moore L, van den Bree MB (2013). A systematic review of the relationships between family functioning, pubertal timing and adolescent substance use. Addiction.

[32] Lopes-Rosa R, Kessler FP, Pianca TG, Guimaraes L, Ferronato P, Pagnussat E, Moura H, Pechansky F, von Diemen L (2017). Predictors of early relapse among adolescent crack users. J Addict Dis.

[33] Acri MC, Gogel LP, Pollock M, Wisdom JP (2012). What adolescents need to prevent relapse after treatment for substance abuse: a comparison of youth, parent, and staff perspectives. J Child Adolesc Subst Abuse.

[34] Kirisci L, Tarter R, Ridenour T, Reynolds M, Horner M, Vanyukov M (2015). Externalizing behavior and emotion dysregulation are indicators of transmissible risk for substance use disorder. Addict Behav.

[35] Dell'osso L, Carmassi C, Stratta P, Massimetti G, Akiskal KK, Akiskal HS, Maremmani I, Rossi A (2013). Gender differences in the relationship between maladaptive behaviors and post-traumatic stress disorder. A Study on 900 L' Aquila 2009 earthquake survivors. Front Psychiatry.

[36] Wang LJ, Chen MY, Lin CY, Chong MY, Chou WJ, You YH, Tsai CP, Chen YS, Lu SF (2018). Difference in long-term relapse rates between youths with ketamine use and those with stimulants use. Subst Abuse Treat Prev Policy.

[37] Poudel A, Gautam S (2017). Age of onset of substance use and psychosocial problems among individuals with substance use disorders. BMC Psychiatry.

[38] McLaughlin A, Campbell A, McColgan M (2016). Adolescent substance use in the context of the family: a qualitative study of young people's views on parent-child attachments, parenting style and parental substance use. Subst Use Misuse.

[39] Lui CK, Sterling SA, Chi FW, Lu Y, Campbell CI (2017). Socioeconomic differences in adolescent substance abuse treatment participation and long-term outcomes. Addict Behav.

